# Inflammatory Pseudotumor Containing Necrotizing Granulomatous Lesions of Kidney: A Hitherto Undescribed Entity

**DOI:** 10.1155/2014/263859

**Published:** 2014-10-14

**Authors:** Tadashi Terada

**Affiliations:** Department of Pathology, Shizuoka City Shimizu Hospital, Miyakami 1231 Shimizu-ku, Shizuoka 424-8636, Japan

## Abstract

Herein reported is a case of inflammatory pseudotumor (IPT) of kidney. It is not described in WHO, AFIP, and other books. A review of the literature revealed about 35 cases. A 76-year-old man underwent nephrectomy under clinical diagnosis of renal pelvic carcinoma. Grossly, a solid tumor was seen in renal parenchyma. Microscopically, it was composed of spindle cell tissue with inflammation and many necrotizing granulomas. Epithelioid histiocytes were abundant but giant cells were few. Lymphocytes and plasma cells were also seen. The features suggested tuberculosis (TB), but Ziehl-Neelsen stains and PCR revealed no TB bacillus. Immunohistochemistry showed that the tumor spindle cells were positive for vimentin, CD68, CD45, and Ki-67 (labeling = 18%), *α*-smooth muscle antigen, and NSE. Focal staining of KIT (mast cells), S100 protein (Langerhans cells), and CD10 (spindle cells) was present. IgG4 was negative. The tumor spindle cells were negative for other antigens examined.

## 1. Introduction

In general, inflammation does not become apparent tumor. However, inflammation rarely forms apparent tumor, and such cases are called inflammatory pseudotumor (IPT). Tuberculosis (TB) rarely manifest as tumors, and such tumors of TB are called tuberculomas. Also, inflammation may lead to neoplasms. IPT is extremely rare and occurs in any organs, particularly in liver and lungs. This entity in kidney is not described in WHO blue book [1] and AFIP series [2]. However, in the world literature, there have been at least 35 cases of renal IPTs [3–11]. All reported cases are single case reports. All reported cases of IPT of kidney showed typical features of IPT including variable proliferations of fibroblasts, myofibroblasts, extracellular collagens, and abundant infiltrations of lymphocytes and plasma cells. Recently, IgG4-related IPTs have been reported sporadically [6, 10]. IgG4 is now well known to be associated with fibrosing inflammations such as sclerosing pancreatitis and cholangitis. The author herein reports a case of IPT of kidney with atypical features including necrotizing granulomatous changes and mild inflammatory infiltrates of lymphocytes and plasma cells.

## 2. Case Report

**Figure 1:**
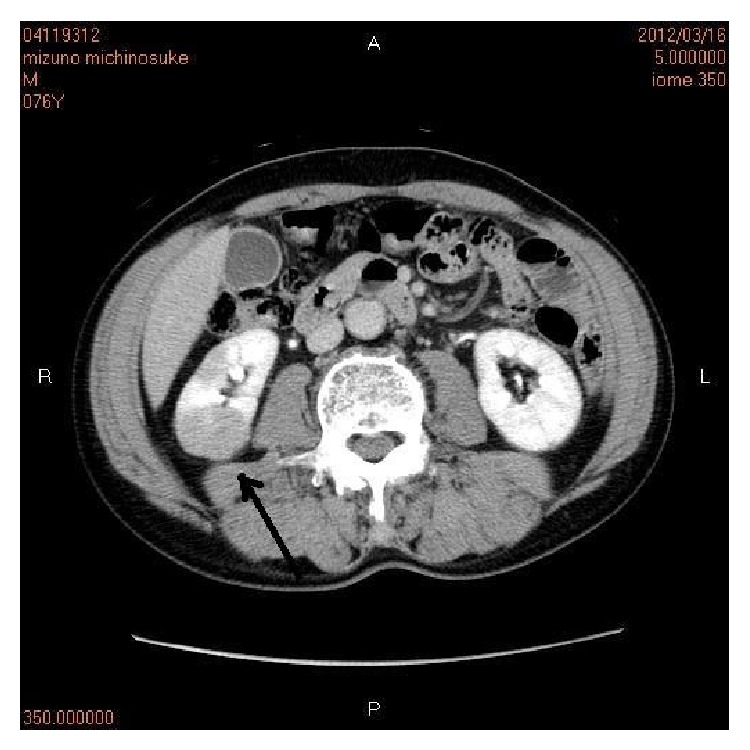
Enhanced CT findings. The right kidney (arrow) shows irregular enhancement compared to the left kidney. The findings suggest renal pelvic carcinoma.

A 75-year-old man presented with dysuria. Imaging revealed prostatic hyperplasia. Blood laboratory test showed mild PSA elevation of 7.6 ng/mL. No infections were seen. Other tumor markers were within normal ranges. Core biopsies of prostate showed no malignant cells. Next, he was found to have small grade 2 papillary urothelial carcinoma with mild invasion (stage pT1) in the bladder, and TUR-BT was performed. One year later, the patient was found to have atypical cells in urine by cytology. Cystoscopy revealed no bladder tumor, but enhanced CT showed irregular shadows in right kidney (Figure 1). The diagnosis of radiologists and urologists was renal pelvic carcinoma, although renal parenchymal tumor was not excluded. The patient underwent open right ureteronephrectomy.

**Figure 2:**
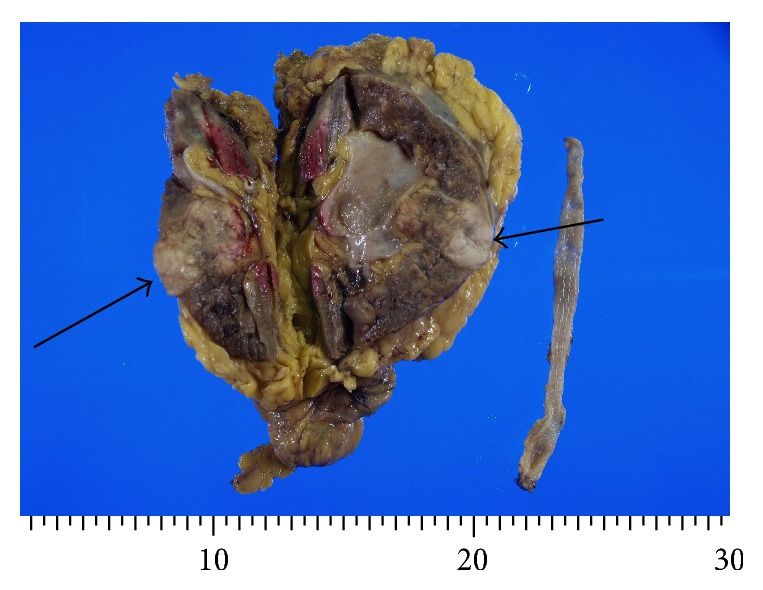
Macroscopic findings of the resected right kidney and ureter. A solid well-demarcated white tumor (arrows) measuring 1.4 × 1.6 × 1.8 cm is seen in the parenchyma. The renal pelvis and ureter show no tumors.

Grossly, the kidney specimens showed a solid white tumor measuring 1.4 × 1.6 × 1.8 cm in renal parenchyma (Figure 2). Renal pelvis was free from tumors. The tumor was well defined from renal parenchyma. Five histological sections were taken from the tumor for microscopy and four sections from nontumorous kidney and pelvis.

**Figure 3:**
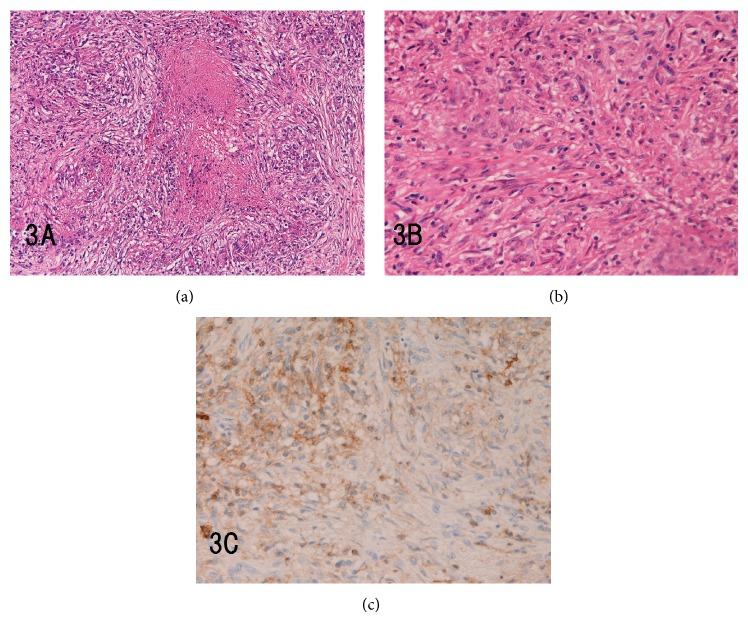
The morphologic findings of the tumor. (a) Low power view. The tumor is composed of spindle cells in which many necrotizing granulomatous lesions are scattered. Inflammatory infiltrations are none or few (HE, ×40). (b) High power view. The tumor is composed of spindle cells (HE, ×200). (c) Immunohistochemical findings of the tumor. The tumor spindle cells are positive for CD68 (×200).

Microscopically, the kidney tumor was well defined from renal parenchyma. The tumor was composed of relatively compact spindle cells tissue with mild inflammations and many necrotizing granulomas (Figures 3(a) and 3(b)). Epithelioid histiocytes were abundant but giant cells were few. A mild degree of lymphocytes and plasma cells were seen. There were no features of dense collagenous, myxoid or vascular areas. The features suggested tuberculosis (TB), but Ziehl-Neelsen (ZN) stains revealed no signals. PCR technique for TB DNA, done twice, revealed no signals.

Scrutiny of body by imaging revealed no evidence of TB, and lungs were free from inflammation and tumor. Upper and lower gastrointestinal endoscopy showed no significant lesions except for a few adenomas of the colorectum. ZN stain and PCR for TB of bronchoalveolar lavage, done twice, showed no evidence of TB infection, and PCR of urine also showed no TB signals. Therefore, TB infection was not likely. No organ culture study related to the renal tumor was carried out because the samples were already fixed in formalin.

Immunohistochemistry, which was performed by envision method [12–15], of tumor showed that the tumor spindle cells were positive for vimentin, CD68 (Figure 3(c)), and Ki-67 (labeling = 18%). Moderate staining of *α*-smooth muscle antigen (ASMA) and neuron specific enolase (NSE) was noted in tumor spindle cells. Focal staining of KIT (mast cells), S100 protein (Langerhans cells), and CD10 (spindle cells) was present. The tumor spindle cells were negative for other antigens. The entrapped tubules were positive for cytokeratin (CK) AE1/3, CK CAM5.2, CK 7, CK18, CA19-9, and MUC1 and entrapped glomeruli for CD10. The vessels of tumor were positive for CD31, factor VIII-related antigen, and CD34. Immunostaining of CD3, CD20, CD38, CD45, CD45RO, CD56, CD57, CD79*α*, *κ*-chain, *λ*-chain, and CD138 revealed a few lymphocytes and plasma cells. Immunostaining of IgG4 showed few IgG4 containing cells. There were no immunoreactions for CK34BE12, CK5/6, CK14, CK8, CK19, CK20, CEA, EMA, p63, p53, ErbB2, chromogranin, synaptophysin, NCAM, MUC2, MUC5AC, MUC6, CDX-2, and myoglobin. The author diagnosed the tumor as IPT because the tumor was inflammatory in nature although histologic features were not typical.

## 3. Discussion

Clinically, the present IPT was diagnosed as renal pelvic carcinoma. This diagnosis was based on urine atypical cells and imaging particularly. In most reported cases of IPT of kidney, renal cell carcinoma (RCC) or urothelial carcinoma of pelvis was suspected clinically [3–10]. This situation is similar to IPT of other organs. Thus, IPT mimics clinically malignant tumor [3–10].

According to previously reported cases [3–11], IPT of the kidney usually includes 3 histological patterns with one pattern being predominant. The three patterns are (a) myxoid, vascular, and inflammatory areas, somewhat reminiscent of nodular fasciitis, (b) compact spindle cell areas admixed with inflammatory cells including lymphocytes, plasma cells, and eosinophils, showing some similarity to fibrous histiocytoma, and (c) dense fibrocollagenous areas of desmoid-like appearance. In the present IPT of kidney, the second area (compact spindle cell areas) was predominantly seen.

The present IPT of kidney showed many necrotizing granulomatous features. Epithelioid histiocytes positive for CD68 and CD45 were seen. However, giant cells were few and no apparent Langerhans giant cells were seen. Since these findings suggested TB, the author examined ZN stain and found no TB bacillus. Further, the author carefully performed PCR, which failed to identify TB. All the data denied TB infection in the present IPT of kidney.

IPT of any locations shows characteristic histologies [3–10]. IPT shows proliferation fibroblasts, myofibroblasts, and infiltrations of lymphocyte and plasma cells. These inflammatory infiltrations are the most characteristic features of IPT. In contrast, the current IPT showed proliferation of vimentin positive fibroblasts and ASMA-positive myofibroblasts. These are compatible with IPT. However, the current renal tumor showed many necrotizing granuloma-like areas and infiltrations of lymphocyte and plasma cells were almost none. Such cases of IPT with unusual morphologies have not been reported, to the best of the author's knowledge. Therefore, the current IPT has not been reported with regard to its morphology and appears as a new entity. Since the histologies of the present IPT seem to be hitherto undescribed in the literatures, text books of WHO and AFIP, and so forth, the author herein reports this case. Lymphocytes and plasma cells were almost absent: the most unusual features of the present tumor.

To characterize the tumor, the author performed extensive immunohistochemical studies. The tumor spindle cells were positive for vimentin, CD68, CD45, and Ki-67 (labeling = 18%). Vimentin positivity indicates that the spindle cells are mesenchymal cells. Positivity of CD68 shows that the epithelioid cells in the necrotizing granulomatous lesions are histiocytes. CD45 positivity was located mostly in the epithelioid cells, suggesting that the epithelioid cells have white blood cell characters and are compatible with authentic epithelioid cells. It was noteworthy that IgG4 was not expressed in the present study. Recently, it was found by the Japanese that that IgG4 may contribute to tissue fibrosis, including sclerosing pancreatitis and cholangitis and IPT [6, 10]. Thus, the present IPT is not associated with IgG4-related diseases.
